# Determinants of community-based health insurance membership renewal decision among rural households in Kellem Wollega zone, Oromia regional state, Ethiopia: a community-based cross-sectional study

**DOI:** 10.3389/fpubh.2023.1192991

**Published:** 2024-01-04

**Authors:** Edosa Tesfaye Geta, Kifile Abdisa Lushe, Adisu Ewunetu Desisa, Dufera Rikitu Terefa, Melese Chego Cheme, Adisu Tafari Shama, Matiyos Lema

**Affiliations:** ^1^Kellem Wollega Zonal Health Department, Dembi Dollo, Ethiopia; ^2^Department of Public Health, Institute of Health Science, Wollega University, Nekemte, Ethiopia

**Keywords:** CBHI scheme, membership renewal decision, rural households, Kellem Wollega, Ethiopia

## Abstract

**Background:**

Despite the fact that community-based health insurance (CBHI) is a promising program to achieve the goal of universal health coverage (UHC), it faces challenges that are not only due to low enrollment but also due to membership renewal decision that impact its sustainability. Hence, the study aimed to identify the determinants of CBHI membership renewal decision among rural households in Kellem Wollega zone, Ethiopia.

**Methods:**

The study was conducted in Kellem Wollega, Ethiopia, among rural households from March 30–April 30, 2022, using a community-based cross-sectional study design. An interviewer-administered structured questionnaire through face-to-face interviews was used. Using a systematic random sampling method, 551 households were selected making 540 (98%) response rates. The data was entered into EPI Data 3.1 and analyzed using SPSS 25 software. Descriptive statistics, binary, and multiple logistic regressions were performed. Using multiple logistic regressions, a significant association between the CBHI membership renewal decision and independent variables was identified, declaring the statistical significance level using a 95% confidence interval (CI) at *p* < 0.05.

**Results:**

The overall rate of CBHI membership renewal decision among households was estimated to be 365 (67.6%, 95% CI = 63.7–71.5%). The factors that significantly influenced the households’ membership renewal decision were family size (AOR = 0.46, 95% CI = 0.25–0.86), low literacy status (AOR = 0.28 95% CI = 0.12–0.64), lower than middle-level of wealth index (AOR = 9.80, 95% CI = 2.75–34.92), premium affordability (AOR = 4.34, 95% CI = 2.08–9.04), unavailability of services (AOR = 0.26, 95% CI = 0.12–0.55), trusting in health facilities (AOR = 5.81, 95% CI = 2.82–11.94), favorable providers’ attitude toward members (AOR = 8.23, 95% CI = 3.96–19.64), good quality of service (AOR = 4.47, 95% CI = 2.28–8.85) and health care seeking behavior (AOR =3.25, 95% CI = 1.32–7.98).

**Conclusion:**

The overall CBHI membership dropout decision rate among rural households was high, which could affect health service provision and utilization. Therefore, the insurance scheme and contracted health facilities should consider and work on family size and wealth status when membership premiums are calculated, the education level of households when creating awareness about the scheme, building trust in the contracted health facilities by providing all promised benefit packages of health services with good quality, and improving the attitude of health care providers towards the scheme members.

## Introduction

Prompt access to health services that include prevention, treatment, rehabilitation, and promotion is essential with the exception of just a small portion of the population; this is not attainable in many countries without a successful system of health financing that determines if an individual can pay to utilize healthcare when necessary. The World Health Organization (WHO) provides recommendations for how countries could modify their financial systems to accelerate the process of moving to UHC while sustaining the progress that has been made so far (1).

Globally, countries have put UHC at the forefront of their health policies and plans while some of them have attained it, the majorities are still working towards UHC, and progress varies across countries (2, 3). Evidence shows that most low-income-countries (LMICs) have not been able to achieve the goals of UHC because of issues with weak public health care systems, building resilient health systems, financing health care and reducing financial risk, epidemiological and demographic issues, governance, and leadership (4).

How to pay for health services is a basic issue that must be addressed. Searching for a way to raise adequate resources is noticeably vital. Even though the major dependence of financial sources for the health system is on public funding, it is vital to guarantee access to healthcare while, at the same time, protecting families from catastrophic medical bills (5).

Consequently, it has been proven that health insurance enables people to feel more financially protected by reducing households’ out-of-pocket medical expenses, catastrophic medical expenses, overall medical expenses, and poverty. Additionally, it protects household savings, assets, and consumption habits in a favorable manner (6).

A recently developed concept called CBHI promises to provide low-income rural households better access to high-quality healthcare and financial protection against the cost of illnesses. Many developing countries presently provide CBHI to their rural populations, and research on the program’s effects on the well-being of the underprivileged in these areas is still ongoing (7).

A CBHI model can only partially assist countries in moving to UHC when it solely relies on small-scale, voluntary programs and small pools with little to no subsidies for the poor and vulnerable populations. This is despite the fact that CBHI is one method of organizing community initiatives (8).

Countries are working on CBHI, where risks are pooled and shared, to improve access to quality care, overcome catastrophic out-of-pocket expenses, and strengthen healthcare finance. To reach this, households could be convinced, enrolled in the insurance, and also renewed the membership, since a crucial aspect of achieving UHC is expanding a financial hazard pooling system that provides cross-subsidization in the health scheme, which is health insurance (9).

The government of Ethiopia introduced the CBHI scheme in 2011 in 13 districts of the four regions with large populations, namely Oromia, Amhara, SNNP, and Tigray, as a pilot project. After evaluating the feasibility and accomplishments of the program in the pilot districts, the government expanded its implementation to other districts. Currently, about 512 districts have implemented it; the aim is to enable the poor to get quality health services regardless of their economic status (10).

The decision to drop the membership also hinders risk pooling and resource mobilization for effective plan management and creates long-term sustainability issues. Only 2 million, or 0.2%, of Africa’s 900 million potential members are enrolled. In sub-Saharan African (SSA) countries, with the exception of Ghana and Rwanda, the membership ratio is below 10% (9–11). In Guinea-Conakry, the initial enrollment rate of CBHI in 1998 was 8%, but this enrollment rate declined to 6% a year later and the main reasons for non-enrollment and dropout were scheme affordability, poor quality of care, and inability to pay the premium (5). Low enrollment, or the decision to drop out, and the presence of too few people in the scheme are endangering the sustainable progress of this reform in many countries, including SSA (12).

The development of the CBHI program faces a great challenge that is not only due to low enrollment but also due to high membership dropout decision rate, which causes the scheme to be unsustainable by decreasing its coverage directly (13, 14). A study conducted in Ethiopia documented that enrollment a year after initiation increased only from 41 to 48%. On the other hand, 18% of those who joined the program in its first year did not renew their membership the year after (14).

**Figure 1:**
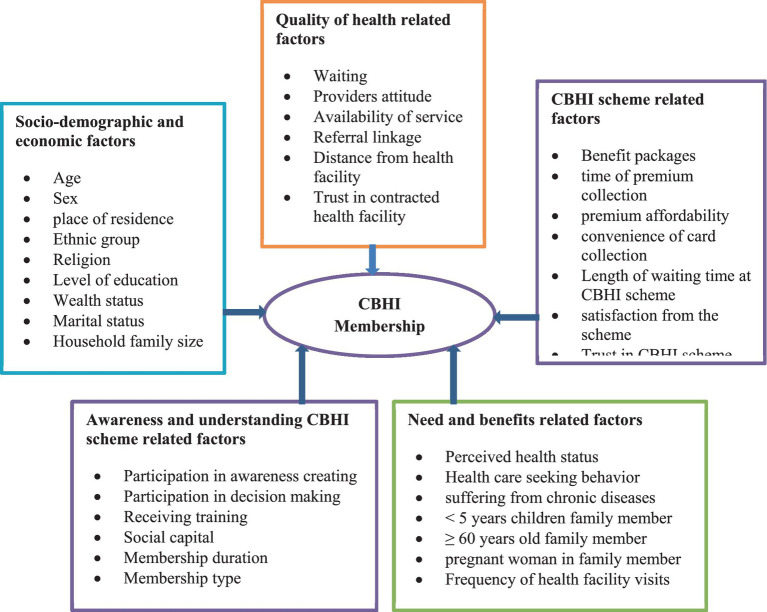
A conceptual framework of determinants of CBHI scheme membership renewal decision.

There are different factors that influence the households’ decision to renew their CBHI scheme either positively or negatively that can be categorized into: socio-demographic and economic related factors, quality of health-related factors, CBHI scheme related factors, awareness and understanding of CBHI scheme related factors, and need and benefit related factors (Figure 1) (15).

In the Kellem Wollega Zone, the CBHI scheme was first implemented in 2011. During the study period, on average, the amount of the premium per household was 450 Ethiopian Birr (ETB) that was 8.67 US dollars. The coverage of CBHI membership was not improved, even though there have been more participants each year in the zone compared to the national average. Reports from the zonal health department showed that the enrolled members were not renewing their memberships on an annual basis in rural areas of the zone (16). Although initial enrollment is important, scheme sustainability clearly requires the decision of members on membership renewal. Hence, the study aimed to identify the determinants of community-based membership renewal decision among rural households in Kellem Wollega Zone, Oromia Regional State, Ethiopia.

## Methods and materials

### Study setting and design

The study was conducted in Kellem Wallaga, Oromia regional state, Ethiopia, among insured rural households from March 30, 2022, to April 30, 2022, using a community-based cross-sectional study design. The zone was one of the 20 zones of the Oromia regional state, which was established in 2007, and it had 12 districts, one of which was urban and the others rural. Dembi Dollo is the capital of the zone, which is located about 652 km west of Addis Ababa, the capital of the country.

The zonal health department’s reports of 2019/2020 showed that six districts were implementing the CBHI scheme, and all of these districts have begun offering health services as part of this program. Seyo and Gidami districts began offering services in 2016, while the four remaining districts namely Dalle Sadi, Dale Wabera, Hawa Galan and Lalo Kile began doing so in 2020. They launched the CBHI in different year; 53.5% of the zone’s population were enrolled in the CBHI. The two pilot districts were selected for this study because the remaining four districts have not yet started the CBHI membership renewal process.

The total population of the Gidami was estimated to be 112,190 and from a total of 7,010 households, only 3,225 (46%) were enrolled in the CBHI program. The community had five government health centers and 22 health posts that provide health services for the community. The total population of the Seyo district was 76,207, and from a total of 4,760 households, only 2,428 (51%) were enrolled in the CBHI program. The district had 6 government health centers and 24 health posts that provide health services for the community, according to the 2019/2020 zonal health department report (16).

### Population and eligibility criteria

All households who were ever enrolled in the CBHI scheme in rural districts of Kellem Wollega Zone were considered as the source population. All households that were registered to the CBHI scheme and found in selected villages of the zone were considered as the study population, whereas the household head who ever enrolled in the CBHI scheme was considered as the study unit.

All households enrolled in the CBHI scheme in selected kebeles of the Kellem Wollega zone were included in the study, whereas all households who were members of the scheme but came from other areas, whose residence was less than 1 year, and those whose enrollment duration less than 1 year were excluded from the study.

### Sample size determination and sampling techniques

Sample size was calculated by using the single population proportion formula, assuming 68.1% of the households made the decision to renew their CBHI membership (17), 95% CI, and a 0.05 margin of error. Since two-stage sampling was used, the 1.5 design effect was considered, $n=\frac{{\left(Z\alpha /2\right)}^{2}p\left(1-p\right)\;}{d^{2}}$, where, *n* = calculated sample size, *z* = 95% CI, d = margin of error at 0.05, and p = proportion of households’ membership renewal decision = 68.1%.

So, *n* = (1.96)^2^ × 0.681(1–0.681)/ (0.05)^2^ = 334, multiplying by 1.5 design effect and adding 10% non-response rate, the final sample size determined was 551.

**Figure 2:**
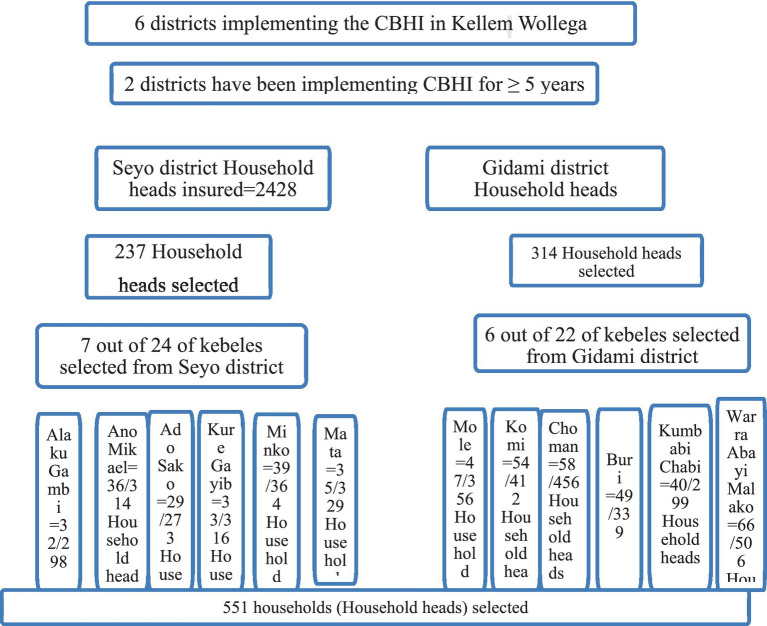
Schematic presentation of sampling procedure.

Since the zone consisted of 12 districts and 256 rural kebeles, and six districts were implementing the CBHI scheme while the other four districts started to implement the scheme in 2020, presumably two districts that started implementing the CBHI scheme before 5 years ago when the study was conducted were selected. Then the required sample size was proportionally allocated to both districts, and by the simple random sampling method, kebeles (villages) were selected from both districts, and 30% of the total kebeles were selected from both districts. Accordingly, 6 kebeles out of 22 kebeles from Gidami district and 7 kebeles out of 24 kebeles from Seyo district were selected (Figure 2).

The lists of households having households or spouses with informal work were obtained from the district administration office (village register), and after getting the lists of households from each selected kebele, samples were selected by systematic random sampling to get the data required. Finally, the study participants were selected using systematic random sampling methods from the CBHI insurance scheme registration book list at every k^th^ household in the frame from each kebele until the sample size of that kebele reached the value shown in this formula: k = N/n. Accordingly, N = study households (5653), and n = calculated sample size (551); then k = 5653/551 = 7, every 7^th^ household was selected from each kebele. The lottery method was used to select the first households from 1 to 7 serial numbers.

### Study variables

Community-based health insurance scheme membership renewal decision was considered as a dependent variable, whereas socio economic and demographic factors (age, sex, occupation, gender, religion, educational status, marital status, household size, wealth index, presence of under five children in households, and presence of the older adult in households), health and health services related factors (accessibility of health facility, availability of health service, utilization of health service, perceived quality of service, chronic disease, and recent illness episode), CBHI-related factors (benefit package, scheme experience, premium collection convenience time, duration of CBHI enrolment, affordability of premium collection, premium collection convenience time and duration of CBHI) (Figure 1).

### Operational definitions

#### Membership renewal decision

Households who had CBHI scheme for more than 1 year and who were still enrolled in the time of the survey, and households that were enrolled in the 1st year of operation, dropped in the 2nd year of operation, and enrolled again in 3rd year of operation were considered as “*1 = Renewed*, otherwise *0 = Dropped*.”

#### Understanding CBHI scheme

The participants were interviewed on 10 items (those who fall sick should consider membership in CBHI, only the very poor who cannot afford to pay can need to join the CBHI, under CBHI, you pay money in order for CBHI to finance your future health care, CBHI programs are like saving schemes; you will receive interest and get your money back, if you do not make claims through CBHI, your premium will be returned, all health care costs are covered by the CBHI program; Cronbach’s Alpha (α) = 0.71). The respondents rated each item using a 3-points Likert’s scale (correct, not correct, I do not know), and finally the overall mean score was calculated, with ≥ mean score categorized as *1 = high level of understanding*, < mean score classified as *2 = low level of understanding*.

#### Trust in CBHI scheme

The participants were interviewed on five items (community is involved in the management of local CBHI scheme, premium contributed by member used for CBHI purpose only, CBHI scheme is providing reimbursement service, the local CBHI management is trust worth, the CBHI scheme distribute ID card as early as members enrolled/return as early as they send for renewal; α = 0.91) using a 5-points Likert’s scale (1 = strongly disagree, 2 = disagree, 3 = neutral, 4 = agree, 5 = strongly agree) and finally the overall mean score was calculated and ≥ mean score categorized as; *1 = Good trust*, <mean score classified as, *2 = Poor trust*.

#### Trust in contracted health facilities

The participants were interviewed on 10 items (health facility provides all services expected to be given at its level, HF always has sufficient health professionals, HF has improved referral system, physical facility is visually clean, attractive and comfortable, health facility staff has sufficient competence to treat patients, HF staff is committed in providing services, HF provides service timely, HF is concerned for the need of CBHI members, and HF reliable in handling patient problems; α = 0.93) using a 5-points Likert’s scale (1 = strongly disagree, 2 = disagree, 3 = neutral, 4 = agree, 5 = strongly agree), and finally the overall mean score was calculated and ≥ mean score categorized as ***1 = good trust***, < mean score classified as ***2 = poor trust***.

#### Provider’s Attitude towards CBHI member

The participant households were interviewed on 10 items (CBHI has a potential on promoting health care seeking behavior from modern health care institutions, CBHI protects house hold from unaffordable health care expenditure, premium payment for CBHI scheme is expensive, CBHI is a means of collecting revenue(profit)to the government, CBHI scheme members receive low quality of services than non-members, mistreatments of patients by the professional is common for members than non-members, I did not have trust in management and administration of CBHI scheme, CBHI is relevant only to promote health condition of the poor, health insurance is good to pool the risk of health expenditure within sick health, and CBHI should be advocate and scaled up to improve health condition of rural community; α = 0.74) using a 5-points Likert’s scale (1 = strongly disagree, 2 = disagree, 3 = neutral, 4 = agree, 5 = strongly agree), and finally the overall mean score was calculated and ≥ mean score categorized as, 1 = favorable < mean score classified as, 2 = unfavorable.

#### Community-based health insurance scheme experience

The participants were interviewed on 6 items (the local CBHI agent tries hard to solve CBHI implementation problems; CBHI members have the right to guide and supervise the activities of CBHI management activities; the CBHI benefits package meets the requirements of my households; I am satisfied with the experience of the local CBHI office when I go to register; I am satisfied with the local CBHI office when I go to pay regular contributions; and members of CBHI are treated the same as non-members; α =0.91) using a 5-points Likert’s scale (1 = strongly disagree, 2 = disagree, 3 = neutral, 4 = agree, 5 = strongly agree), and finally, the overall mean score was calculated and ≥ mean score categorized as, 1 = good experience, < mean score classified as, 2 = poor experience.

#### Quality of health services at contracted health facilities

The households were interviewed on four items (availability of adequate drugs, availability diagnostic/laboratory services, improvement in waiting time to get services, and improvement in referral system; α = 0.82) using a 5-points Likert’s scale (1 = strongly disagree, 2 = disagree, 3 = neutral, 4 = agree, 5 = strongly agree), and finally the overall mean score was calculated and ≥ mean score categorized as 1 = good quality, < mean score classified as, 2 = poor quality.

#### Affordability of the premium

The participant households were interviewed on four items (time interval of premium payment is convenient for my household, the CBHI registration fee is easily affordable, the CBHI regular contribution is easily affordable, and received promised benefit package during membership; α = 0.801)using a 5-points Likert’s scale (1 = strongly disagree, 2 = disagree, 3 = neutral, 4 = agree, 5 = strongly agree), and finally the overall mean score was calculated and ≥ mean score categorized as: 1 = affordable, < mean score classified as: 2 = unaffordable.

#### Household wealth index

Households’ asset data was collected based on the kinds of assets they owned. Then factor scores were derived using principal component analysis (PCA), and based on the composite scores of the wealth index, the households were categorized in to five equal groups (quintiles) based on their rank wealth status. Accordingly, *1 = Extremely poo*r: the 1st quintile(0–20% of the range), *2 = Poo*r: the 2nd quintile (20–40% of the range), *3 = Middle leve*l: the 3rd quintile (40–60% of the range), *4 = Rich:* the 4th quintile(60–80% of the range), and *5 = Very ric*h: the 5th quintile (80–100% of the range).

### Data collection tool and procedure

An interviewer-administered structured questionnaire that was adapted from related articles (12–14, 17) to collect relevant information. The questionnaire had five parts: socio-economic and demographic characteristics; CBHI information; individual or household-related factors; CBHI-related factors; and health service use-related factors.

Data was collected by six BSc-trained health professionals using the pre-tested structured questionnaire through face-to-face interviews. Two supervisors who were qualified with a master’s degree in public health were recruited. The data collectors and supervisors were trained for 1 day on the objectives of the study, the data collection tool, approach to the interviews, details of interviewing techniques, respect, and maintaining the privacy and confidentiality of the respondents.

### Data processing and analysis

After the data collection from each selected household, the data was entered into EPI data version 3.1 and analyzed using SPSS 25 software. Hosmer-Lemeshow goodness-of-fit was used to check the fitness of a multi-variable logistic regression model that had been eventually fitted, the *p*-value was found to be 0.712. Descriptive statistics, including frequencies, cross-tabulation, averages, and percentages, were performed. Binary and multiple logistic regressions were used to identify a significant association between the CBHI membership renewal decision and independent variables. Using multiple logistic regressions, a significant association between the CBHI membership renewal decision and independent variables was identified, with the statistical significance level using 95% CI at *p* < 0.05.

### Data quality management

The internal consistency of items for independent variables measured in Likert’s scale was checked by Cronbach’s alpha. The questionnaire was prepared in English and translated from English to Afan Oromo. Before the actual data collection, pre-testing was done by the data collectors on 28 individuals (5% of the sample size) in Dalle Sadi district, Kellem Wollega Zone, and appropriate modifications were made after analyzing the pre-test results. To ensure data quality, the principal investigators provided training all data collectors and supervisors. All collected data were checked for completeness and consistency by the supervisors every day, and onsite close supervision and technical support were given by supervisors and the principal investigators. During the data collection period, when the household head was unavailable, the data collectors asked the house member who could respond to the interview or when he/she would be available and returned to home in the next day for interview.

### Ethical considerations

Ethical clearance (*Ref: WU/RD/526/2014*) was obtained from the Research Ethics Review Committee (*RERC*) at Wollega University. A formal letter that explained the objectives, significance, and expected outcomes of the study was written to Kellem Wollega zonal health department from the Wollega University, Institute of Health Sciences and requested cooperation. Based on a supportive letter from Wollega University, Kellem Wollega zonal health department issued a support letter to the districts Health Office.

All study participants were well informed about the purpose of the study, and informed written consent was secured from the study participants prior to the interview. The study participants’ confidentiality was maintained, and no personal identifiers were used in the data collection tools; instead, codes were used in their place. All paper-based and computer-based data were kept in protected and safe locations. The recorded data were not accessed by a third party except the research team, and data sharing will be enacted based on the ethical and legal rules of data sharing.

## Results

### Socio-demographic and economic characteristics of the households

A total of 540 participants responded to the interview, making the response rate 98%. The mean age of the respondents was 45.14 ± 10.84 years. Majority of the respondents, 494 (91.5%), were males, and 506 (93.7%) of the respondents were married. One third, 174 (32.2%) of the participants were unable to read and write. More than three-fourths, 472 (87.5%) of the study participants were farmers. About 250 (46.3%) of the households had fewer than five members in their household. The wealth status of the households was ranked. As a result, 108 (20%), 107 (19.8%), 109 (20.2%), 111 (20.6%), and 105 (19.4%) were extremely poor, poor, middle level, rich, and very rich, respectively (Table 1).

**Table 1:** Socio-demographic and economic characteristics of the households in Kellem Wollega Zone, Oromia regional state, Ethiopia 2022.

Variables n = 540	Response category	Frequency (%)	CBHI membership renewal decision	
			Renewed (%)	Dropped (%)
Sex of Households	Male	494 (91.5)	334 (61.9)	160 (29.6)
	Female	46 (8.5)	31 (5.7)	15 (2.8)
Age	18–30	55 (10.2)	33 (6.1)	22 (4.1)
	31–40	147 (27.2)	103 (19.1)	44 (8.1)
	41–50	151 (28)	95 (17.6)	56 (10.4)
	50+	187 (34.6)	134 (24.8)	53 (9.8)
Religion	Protestant	314 (58.1)	210 (38.9)	104 (19.3)
	Orthodox	99 (18.3)	59 (10.9)	40 (7.4)
	Muslim	127 (23.5)	96 (17.8)	31 (5.7)
Ethnic group	Oromo	517 (95.7)	349 (64.6)	168 (31.1)
	Amhara	23 (4.3)	16 (3)	7 (1.3)
Marital status	Married	506 (93.7)	348 (64.4)	158 (29.3)
	Divorced	11 (2)	7 (1.3)	4 (0.7)
	Widowed	19 (3.5)	8 (1.5)	11 (2)
	Single	4 (0.8)	2 (0.4)	2 (0.4)
Family size	<5 members	250 (46.3)	153 (28.3)	97 (18)
	≥5 members	290 (53.7)	212 (39.3)	78 (14.4)
Presence of <5 years children	Yes	412 (76.3)	276 (51.1)	136 (25.2)
	No	128 (23.7)	89 (16.5)	39 (7.2)
Presence of Old age (60+)	Yes	224 (41.5)	162 (30)	62 (11.5)
	No	316 (58.5)	203 (37.6)	113 (20.9)
Presence of Pregnant woman	Yes	226 (41.9)	145 (26.9)	81 (15)
	No	314 (58.1)	220 (40.7)	94 (17.4)
Education status	Unable to read and write	174 (32.2)	94 (17.4)	80 (14.8)
	Only able to read and write	105 (19.4)	68 (12.6)	37 (6.9)
	Primary education	158 (29.3)	117 (21.7)	41 (7.6)
	Secondary education+	103 (19.1)	86 (15.9)	17 (3.1)
Occupation	Farmer	472 (87.5)	324 (60.1)	148 (27.4)
	Merchant	44 (8.1)	24 (4.4)	20 (3.7)
	Daily laborer	24 (4.4)	17 (3.1)	7 (1.3)
Wealth status	Extremely poor	108 (20)	65 (12)	43 (8)
	Poor	107 (19.8)	80 (14.8)	27 (5)
	Middle	109 (20.2)	81 (15)	28 (5.2)
	Rich	111 (20.6)	71 (13.1)	40 (7.4)
	Very rich	105 (19.4)	68 (12.6)	37 (6.8)

### Sources of information about CBHI scheme

The participants were interviewed about the source of information they heard about CBHI for the first time before they became members. Accordingly, about 344 (63.7%) of them heard about the scheme from officials in public meetings, followed by health professionals in health facilities, which accounted for 164 (30.4%), during CBHI house-to-house awareness creation campaigns, 22 (4.1%), mass media (7.3%), and from neighbors or friends (0.6%).

### Health status and health care seeking behavior

The study assessed the health status and health care seeking behavior in the last 12 months among households (Table 2). As a result, three-fourths of the participants’ members, 383 (75.2%), were free from chronic diseases. The participants also rated their family members’ health status. Accordingly, the majority of them 402(74.4%) rated it as good, whereas only 138 (25.6%) rated it poor. About 428 (79.3%) household members have been ill during the last 12 months and 405 (75%) of them have sought medical treatment from the contracted health facilities. Out of 405 household members, half of them, 203(50.1%) visited a contracted health facilities only once, whereas only 19(4.7%) visited the facility more than four times in the last 12 months.

**Table 2:** Health status and health care seeking behavior among households in Kellem Wollega Zone, Oromia regional state, Ethiopia 2022.

Variables *N* = 540	Response categories	Frequency (%)	CBHI membership renewal decision	
			Renewed (%)	Dropped (%)
Perceived health status	Good	402 (74.4)	280 (51.9)	122 (22.6)
	Poor	138 (25.6)	85 (17.5)	53 (9.8)
Chronic disease in family	Yes	157 (29.1)	90 (16.7)	67 (12.4)
	No	382 (70.9)	275 (50.9)	10 (20)
Ill during last 12 months	Yes	428 (79.3)	288 (53.3)	140 (25.9)
	No	112 (20.7)	77 (14.3)	35 (6.5)
Seeking health care in past 12 months	Yes	405 (75)	258 (47.8)	147 (27.2)
	No	135 (25)	107 (19.8)	28 (5.2)
Frequency of Health facility visit in past 12 months (*N* = 405)	Once	203 (50.1)	133 (32.8)	70 (17.3)
	Twice	135 (33.3)	88 (21.7)	47 (10.6)
	Three times	48 (11.9)	38 (9.4)	10 (2.5)
	Four times and above	19 (4.7)	13 (3.2)	6 (1.5)

### Community-based health insurance membership renewal decision

**Figure 3:**
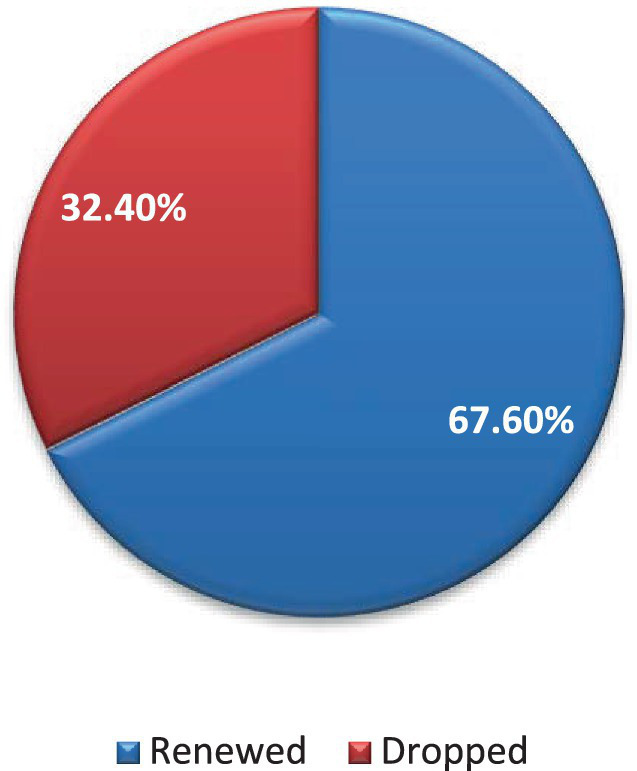
CBHI membership renewal decision among rural households in Kellem Wollega, Oromia regional state, Ethiopia, 2022.

The decision made by households to renew their CBHI membership has also been evaluated by the study. As a result, the majority of households 365 (67.6%, 95% CI = 63.7–71.5%) decided to renew their membership and did so on an annual basis as per to CBHI scheme membership renewal schedule (Figure 3).

Among the study participants, 450 households (83.3%) were payers, and 90 (16.7%) of them were the poorest, exempted from the scheme’s contributions. Almost all of them, 479 (88.7%), had CBHI identification cards. Of those who decided to renew their membership, only 19 (5%) had done so consistently over the past 5 years. Among the 175 households that decided to drop their membership, 153 (87.4%) and 22 (12.6%) of them had stayed in the scheme for one to 3 years and 4 years, respectively. About 24 (4.4%) and 125 (23.1%) of those who renewed and dropped their membership, respectively, had decided not to renew their membership in the next coming year (Table 3).

**Table 3:** Community-based health insurance scheme status among rural households in Kellem Wollega, Oromia regional state, Ethiopia, 2022.

Variables	Response categories	CBHI membership renewal decision		Total (%)
		Renewed (%)	Dropped (%)	
Type of membership	Payer	291	159	450 (83.3)
	Indigent	74	16	90 (16.7)
Having CBHI identification card	Yes	349	130	479 (88.7)
	No	45	16	61 (11.3)
Frequency of membership renewal decision	1 time	33	21	54 (10)
	2 times	84	40	124 (23)
	3 times	156	69	225 (41.7)
	4 times	73	34	107 (19.8)
	≥5 times	19	11	30 (5.5)

**Figure 4:**
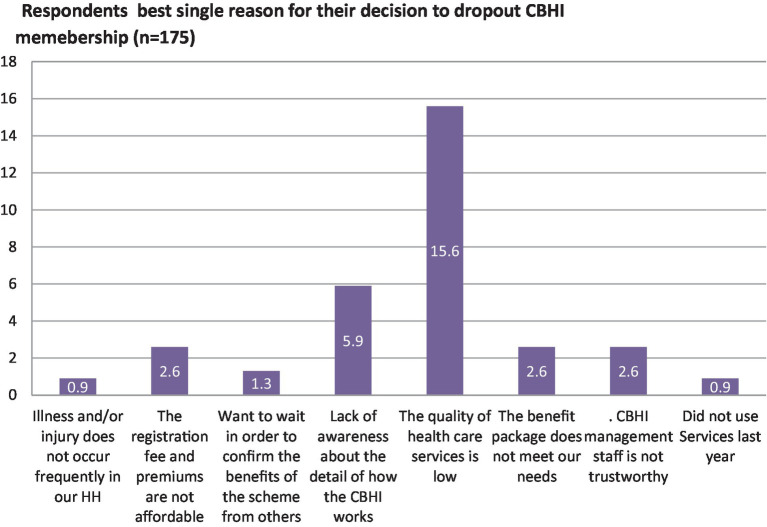
The single best reason for the decision made to drop out of the CBHI scheme membership among households in Kellem Wollega zone, Oromia region, Western Ethiopia, in 2022.

From a total of 175 (32.4%) who decided for the first time to drop their membership, more than one-third of them, 69(39.5%) made their decision after 2 years of membership, followed by 57 (32.6%), 27 (15.4%) after 1 year, 19 (10.9%) after 4 years, and 3 (1.7%) after 5 years. These households reasoned out their best single reason why they decided to drop their membership, such as the quality of health services being low 84(48%), followed by a lack of awareness about the details of how the CBHI works, 32(18.3%) (Figure 4).

More than half of the households 298(55.2%) well understood about CBHI, and the majority of them 222(74.5%) renewed their membership, whereas 242 (or 44.8%) did not understand the scheme well. Among them, 143(59.1%) decided to renew their membership. Among the 316 households (58.5%) who had a favorable attitude toward the CBHI scheme, 231 (73.1%) decided to renew their membership, while 224 (41.5%) had an unfavorable attitude toward the scheme, among whom 134 (59.82%) renewed their membership.

The majority, 344 (63.7%) of the households, did not trust in the CBHI scheme, and 150 (43.6%) of them did not renew their membership, but only 25 (7.9%) of the households that trusted in the CBHI scheme decided to drop their membership and dropped it. More than half, 297 (55%), of the households trusted in the contracted health facilities, and only 33 (11.1%) decided to drop their membership, whereas among those who had no trust in the facilities, 243 (49.5%), with the majority of them, 142 (58.4%), decided to drop their membership. More than half the households, 311 (57.6%) could not afford the premium, and 144 (46.3%) of them dropped their membership, whereas only 31 (13.5%) of the households for whom the premium was affordable decided to drop their membership.

About 251 (46.5%) of the households were members for more than 4 years, among whom only 63 (25.1%) decided to drop their membership, whereas 289 (53.5%) were members for 3 years, among whom 112 (30.8%) dropped their membership. More than half of the households, 304(56%) rated the quality of health services provided by contracted health facilities as good, and the majority of them 226 (74.3%) renewed their membership. The three-fourth of households, 324(60%) had the experience of leaving the contracted health facilities without getting treatments; among them, 138 (42.6%) decided to drop their membership (Table 4).

**Table 4:** Factors influencing CBHI membership renewal decision among households in Kellem Wollega zone, Oromia regional state, Ethiopia, 2022.

Variables (*N* = 540)	Response category	Frequency (%)	CBHI membership renewal decision	
			Renewed (%)	Dropped (%)
Level of understanding CBHI	High	298 (55.2)	222 (41.1)	76 (14.1)
	Low	242 (44.8)	143 (26.5)	99 (18.3)
Attitude toward CBHI	Favorable	316 (58.5)	231 (42.8)	85 (15.7)
	Unfavorable	224 (41.5)	134 (24.8)	90 (16.7)
Trust in CBHI scheme	Good	196 (36.3)	171 (31.7)	25 (4.6)
	Poor	344 (63.7)	194 (35.9)	150 (27.8)
Trust in contracted health facilities	Good	297 (55)	264 (48.9)	33 (6.1)
	Poor	243 (45)	101 (18.5)	142 (26.3)
Premium affordability	Affordable	229 (42.2)	198 (36.7)	31 (5.7)
	Unaffordable	311 (57.6)	167 (30.9)	144 (26.7)
Benefit package as expected received	As expected,	407 (75.4)	292 (54.1)	115 (21.3)
	Below expected	133 (24.6)	73 (13.5)	60 (11.1)
CBHI Scheme experience	Good	293 (54.3)	248 (45.9)	45 (8.3)
	Poor	247 (45.7)	117 (21.7)	130 (24.1)
Length of CBHI enrollment	1–3 years	289 (53.5)	117 (32.8)	112 (20.7)
	≥4 years	251 (46.5)	188 (34.8)	63 (11.7)
Perceived quality of health service	Good	304 (56.3)	226 (41.9)	78 (14.4)
	Poor	236 (43.7)	139 (25.7)	97 (18)
Providers’ attitude toward CBHI members	Favorable	239 (44.3)	213 (39.4)	26 (4.8)
	Unfavorable	301 (55.7)	152 (28.1)	149 (27.6)
Distance of health facilities in minutes	≤ 30 min	169 (31.3)	113 (20.9)	56 (10.4)
	30–60 min	318 (58.9)	224 (41.5)	94 (17.4)
	≥60 min	53 (9.8)	28 (5.2)	25 (4.6)
Leaving health facilities without getting service	Yes	216 (40)	179 (33.1)	37 (6.9)
	No	324 (60)	186 (34.4)	138 (25.6)

### Determinants of CBHI membership renewal decision

In this study, the factors affecting the decision-making process for membership renewal were identified (Table 5). Accordingly, households with fewer than five members were 54% less likely to renew their membership (AOR = 0.46, 95% CI = 0.25–0.86). The education level of households also had a significant influence on decision-making on renewal of membership; as a result, the households whose education level was only being able to read and write were 72% less likely to renew their membership compared to those who attended secondary school or above (AOR = 0.28, 95% CI = 0.12–0.64). On the other hand, households with a lower than middle level of wealth index had a higher likelihood than wealthy households of deciding to renew their membership. As a result, the extremely poor (AOR = 1.71, 95% CI =2.37–12.35), poor (AOR = 5.44, 95% CI = 1.9–32.53), and middle-level (AOR = 9.80, 95% CI = 2.75–34.92) households were more likely to renew their membership compared to the very rich households.

**Table 5:** Determinants of CBHI membership renewal decision among households in Kellem Wollega zone, Oromia regional state, Ethiopia, 2022.

Variables	Response category	Membership renewal decision		Odds ratio, 95% CI	
		Renewed (%)	Dropped (%)	COR	AOR
Sex of households	Male	334 (61.9)	160 (29.6)	1.01 (0.53–1.92)	1.53 (0.51–4.65)
	Female	31(5.7)	15(2.8)	Ref.	Ref.
Family size	<5	153(28.3)	97(18)	0.58 (0.40–0.84)	0.46 (0.25–0.86)*
	≥5	212 (39.3)	78 (14.4)	Ref.	Ref
Old age in family members	Yes	162 (30)	62 (11.5)	1.45 (1.00–2.11)	1.32 (0.69–2.54)
	No	203 (37.6)	113 (20.9)	Ref.	Ref
Under 5 in family members	Yes	276 (51.1)	136 (25.2)	0.89 (0.58–1.37)	0.63(0.29–1.28)
	No	89 (16.5)	39 (7.2)	Ref.	Ref
Education status of households	Unable to read and write	94 (17.4)	80(14.8)	0.23(0.13–0.42)	0.09(0.03–0.25)*
	Only able to read and write	68 (12.6)	37 (6.9)	0.36 (0.19–0.70)	0.28 (0.12–0.64)**
	Primary education	117 (21.7)	41 (7.6)	0.56 (0.30–1.06)	0.40 (0.12–1.27)
	Secondary +	86 (15.9)	17 (3.1)	Ref.	Ref
Wealth status	Extremely poor	65 (12)	43 (8)	0.82 (0.47–1.43)	1.71 (2.37–12.35)**
	Poor	80 (14.8)	27 (5)	1.61 (0.89–2.92)	5.44 (1.9–32.53)*
	Middle	81 (15)	28 (5.2)	1.57 (0.88–2.83)	9.80 (2.75–34.92)**
	Rich	71 (13.1)	40 (7.4)	0.97 (0.55–1.69)	2.35 (0.88–7.05)
	Very rich	68 (12.6)	37 (6.8)	Ref.	Ref.
Understanding CBHI level	High	222 (41.1)	76 (14.1)	2.02 (1.40–2.91)	1.52(0.76–3.04)
	Low	143 (26.5)	99 (18.3)	Ref.	Ref
Attitude to CBHI scheme	Favorable	231 (42.8)	85 (15.7)	1.83 (1.28–2.63)	1.57(0.79–3.09)
	Unfavorable	134 (24.8)	90 (16.7)	Ref.	Ref
Trust in CBHI scheme	Good	171 (31.7)	25 (4.6)	5.29 (3.30–8.47)	1.94 (0.61–6.18)
	Poor	194 (35.9)	150 (27.8)	Ref.	Ref
Premium affordability	Affordable	198 (36.7)	31 (5.7)	5.51 (3.55–8.55)	4.34 (2.08–9.04)***
	Unaffordable	167 (30.9)	144 (26.7)	Ref.	Ref
CBHI Scheme experience	Good	248 (45.9)	45 (8.3)	6.12 (4.09–9.17)	1.75 (0.74–4.15)
	Poor	117 (21.7)	130 (24.1)	Ref.	Ref.
Chronic disease in family	Yes	90(16.7)	67(12.4)	1.90 (1.29–2.79)	0.91 (0.44–1.89)
	No	275(50.9)	10(20)	Ref.	Ref.
Leaving HFs without treatment	Yes	179 (33.1)	37 (6.9)	0.28(0.18–0.42)	0.26(0.12–0.55)***
	No	186 (34.4)	138 (25.6)	Ref.	Ref.
Trust in contracted HFs	Good	264 (48.9)	33 (6.1)	11.25(7.22–17.51)	5.81(2.82–11.94)***
	Poor	101 (18.5)	142 (26.3)	Ref.	Ref.
Providers’ attitude toward members	Favorable	213 (39.4)	26 (4.8)	8.03 (5.04–12.79)	8.23 (3.96–19.64)***
	Unfavorable	152 (28.1)	149(27.6)	Ref.	Ref.
Perceived quality of health service	Good	226 (41.9)	78 (14.4)	2.02 (1.40–2.91)	4.47 (2.28–8.85) ***
	Poor	139 (25.7)	97 (18)	Ref.	Ref.
Traveling time to HF	≤ 30 min	226 (41.9)	109 (20.2)	0.99 (0.95–1.02)	0.41 (0.23–1.83)
	˃30 min	139 (25.7)	66 (12.2)	Ref.	Ref.
Perceived family health status	Good	280 (51.9)	122 (22.6)	1.43 (0.96–2.14)	2.29 (0.99–5.23)
	Poor	85 (17.5)	53 (9.8)	Ref.	Ref.
Seeking health care in past 12 months	Yes	258 (47.8)	147 (27.2)	2.18 (1.37–3.46)	3.25 (1.32–7.98)**
	No	107 (19.8)	28 (5.2)	Ref.	Ref.
Length of CBHI enrolment	1–3 years	117 (32.8)	112 (20.7)	1.90 (1.30–2.74)	2.72 (1.45–5.11)
	≥4 years	188 (34.8)	63 (11.7)	Ref.	Ref.

*p-value < 0.05; **p-value < 0.01; ***p-value < 0.001; Ref, reference; CI, Confidence interval; HF, Health Facility; COR, Crude odds ratio; AOR, Adjusted odds ratio.

Those households who perceived the annual premium as affordable compared with the expected and promised benefits from the CBHI scheme were four times more likely to decide to renew their membership compared to those who perceived the premium as unaffordable (AOR = 4.34, 95% CI = 2.08–9.04). The households that experienced leaving the contracted health facilities without getting treatments because of the unavailability of diagnosis services and drugs in the facilities in the last 12 months were 74% less likely to renew their membership compared to their counterparts (AOR = 0.26, 95% CI = 0.12–0.55).

The trust among households in contracted health facilities was another significant factor. As a result, those households that trusted in the health facilities were almost six times more likely to renew their membership compared to those that had no trust in the facilities (AOR = 5.81, 95% =2.82–11.94). The households were asked their perceptions of the attitude of health care providers toward the scheme members during health care seeking and health service provision. Accordingly, those households that perceived the health care providers as having a favorable attitude toward the scheme members were eight times more likely to renew their membership, compared to their counterparts (AOR = 8.23, 95% CI = 3.96–19.64).

In addition, those households that perceived the quality of health services provided by the contracted health facilities as good were almost five times more likely to renew their membership compared to those who perceived the quality of health care was poor (AOR = 4.47, 95% CI = 2.28–8.85). Those households whose family members sought health care from the contracted health facilities in the past 12 months were three times more likely to decide to renew their membership compared to those households that did not (AOR = 3.25, 95% CI = 1.32–7.98).

## Discussion

Countries all around the world are aiming to increase the number of people who may join the CBHI program and to keep these people enrolled in it. Yet, the majority of LMICs still have considerable difficulties in retaining membership (18). The Ethiopian CBHI scheme is also facing similar problems, like households’ decision to drop their membership. Hence, the study aimed to identify determinants of CBHI scheme membership renewal decision among rural households.

As a result, two-third of the scheme members renewed their membership and decided to continue being members. The results were consistent with those of studies conducted in Ethiopia; Jimma Zone (68.1%) (17) and Dera District (62.8%) (19). However, it was greater than study results from Burkina Faso (45.7%) (20), and lower than the CBHI membership renewal rate in the Gedeo Zone, Southern Ethiopia, which was 82.68% (21), in Ethiopia, 82% of those households who enrolled in the first year renewed their subscriptions during the pilot CBHI scheme and Vietnam (78.9%) (22). This findings discrepancy could have happened due to the study population’s socio-demographic and economic characteristics varying during the study period. In addition, the benefit packages provided by the contracted health institutions may vary from country to country in type, volume, and quality of the health services, membership premium load, and health seeking behaviors among the population. These points have been addressed by the current study.

The households with fewer than five family members were less likely to decide to renew their membership than their counterparts; households with fewer than five members were 54% less likely to do so. Similarly, findings from systematic review studies in LMICs (23), the West Gojjam Zone, Ethiopia (24), and Jimma, Ethiopia (17) showed that households with fewer family members had lower probabilities of renewing their CBHI memberships. This might be explained by the fact that getting health care by directly paying from OOP could be more difficult for large households. Hence, they could prefer to be members of the CBHI scheme for longer by renewing their membership.

The study also showed that the level of education significantly influenced the membership renewal decision-making among households, and the households whose education level was only able to read and write were 72% less likely to renew their membership compared with those who attended secondary school and above. This result was consistent with research from Jimma, Ethiopia (17), Sudan, and Bangladesh (25, 26), which found that insurance members with elementary or secondary education or above were more likely to decide to renew their membership than those without a formal education. Similar to this, in India, the study found that household head education affects membership renewal and that secondary education is linked to a 15%-point rise in renewal (27). Another systematic review study in LMICs found that households with higher levels of education were more likely to renew their participation in the plan than their less educated counterparts (15). One of the potential explanations might be that more educated individuals make timely renewal decision and have a better understanding of the benefit packages and operating principles of the CBHI program.

Households whose wealth status was ranked poor or middle-level were almost ten times more likely to decide to renew their membership. It was supported by the study report in Gurage Zone, Ethiopia, which revealed that those households with the highest wealth status were about two times more likely to drop their membership (28). This could be due to the fact that relatively rich households might purchase more goods and services, including health services that are included in the CBHI service package, and might seek medical care as soon as possible from private health care facilities. In contrast to this, the reports from the study conducted in Ghana (12) and Uganda (29) showed that rich households were more likely to decide to renew membership. This disparity could be due to different levels of understanding of the benefits of the CBHI scheme among members, regardless of their wealth status.

Those households who perceived the annual premium as affordable compared with the expected and promised benefits from the CBHI scheme were four times more likely to decide to renew their membership compared to those who perceived the premium as unaffordable. This finding was supported by evidence from the study conducted in Ethiopia (21), which indicated the odds of CBHI scheme membership renewal were about twelve times higher among households for whom the annual premium was affordable compared to their counterparts. The finding was consistent with the study evidence in Rwanda (30) and Ghana (22). This could be explained by the fact that if the scheme contribution was affordable to the members, households with low income or poor wealth status would be able to pay and would be more likely to retain their CBHI scheme membership.

The households that experienced leaving the contracted health facilities without treatment because of the unavailability of diagnosis services and drugs in the facility in the last 12 months were 74% less likely to renew their membership compared to their counterparts. Similarly, the study report in Ethiopia showed that the scheme members who did not get the prescribed care in contracted health facilities were less likely to renew their membership (28), and the study in Bangladesh revealed that the frequent exposure of household members to the contracted health facilities had an association with membership renewal (31). Similarly, the evidence from a meta-analysis in LMICs revealed that when the promised benefit packages are not fulfilled, the members are more likely to drop their membership (23).

The trust among households in contracted health facilities was another significant factor. As a result, those households that trusted in the contracted health facilities were almost six times more likely to renew their membership compared to those that had no trust in the facilities. It was in line with the study findings from Ethiopia (17, 32–34) and Cambodia (35), which revealed that when households trusted in contracted health facilities, they were more likely to renew their membership.

The households were asked their perceptions of the attitudes of health care providers toward the scheme members. Accordingly, those households that perceived the health care provider as having a favorable attitude toward the scheme members were eight times more likely to renew their membership. Similarly, the evidence from the study conducted in Ethiopia (17) showed that when the health care providers’ attitude toward the scheme members is unfavorable, the odds of a decision to drop their membership could be significantly higher compared to those with favorable attitudes. A study in Ghana also revealed that 30% of the members decided to leave the scheme because of unfavorable providers’ attitudes toward the scheme members (36). This could happen when the demanded health care is provided in a discriminatory manner between the service fee payers and the scheme members when they visit the contracted health facilities.

Those households that perceived the quality of health services provided by the contracted health facilities as good were almost five times more likely to renew their membership compared to their counterparts. This was in line with the study report in Ethiopia, which revealed households who perceived poor health care quality were 12 times more likely to decide to drop their membership (28).

Those households whose family members sought health care from the contracted health facilities in the past 12 months were three times more likely to decide to renew their membership compared to those households that did not. Similarly, the study conducted in Ethiopia showed that the households that had a history of illness and sought medical care were more likely to be enrolled in the CBHI scheme and decide to renew their membership (37), whereas the households that did not experience illness were less likely to renew their membership (28). This could be the case when household members experienced illness during their membership; they might seek and utilize health care from the contracted health facilities compared to those who did not experience illness and may decide to renew their scheme membership.

### Implications of the study

The current study indicated that the membership dropout decision was substantial, which might significantly affect the population coverage rate of CBHI and challenge its sustainability. In addition, the study findings implied that households with a larger family size, lower than middle-level wealth status, and above the primary level education of the household heads, the affordability of the scheme premium to renew membership, the availability of the promised benefit packages of health services with good quality in contracted health care facilities, trusting in the contracted health care facilities, good attitude of health care provides towards the scheme members, and health care-seeking behavior when the households’ members experience health problems were the significant responsible factors that positively influenced the membership renewal decision among rural households to continue their CBHI scheme membership. As a result, the study findings also assist health planners and decision-makers in developing an appropriate plan and strategy to sustain the CBHI scheme in order to achieve UHC, with a focus on how to maintain membership after enrollment taking into account the identified determinant factors.

### Limitations of the study

Despite the fact that the study well addressed the determinants of CBHI scheme membership renewal decision among rural households, it could have a recall and social desirability bias because the respondents were interviewed about the events that occurred in the past 12 months. Furthermore, the current study did not address the potential adverse selection bias that might impact the decision to renew scheme membership.

## Conclusion

The overall CBHI membership dropout decision rate among rural households was high, compared to other study findings that could affect the health service provision and utilization. Therefore, the insurance scheme and contracted health facilities should consider and work on family size and wealth status when membership premiums are calculated, the education level of households when creating awareness about the scheme, building trust in the contracted health facilities by providing all promised benefit packages of health services with good quality, and improving the attitude of health care providers towards the scheme members.

## Data availability statement

The raw data supporting the conclusions of this article will be made available by the authors, without undue reservation.

## Ethics statement

The studies involving humans were approved by Wollega University Research Ethics Review Committee (RERC). The studies were conducted in accordance with the local legislation and institutional requirements. The participants provided their written informed consent to participate in this study.

## Author contributions

EG, KL, AD, DT, MC, AS, and ML participated in developing the study concept and design of the study. EG contributed to the data analysis, interpretation, report writing, manuscript preparation, and acted as the corresponding author. KL contributed to developing the data collection tools, data collection, and data entry to statistical software. AD, DT, MC, AS, and ML contributed to the data collection tool, data collection supervision, and report writing. All authors contributed to the article and approved the submitted version.

## References

[1] WHO (2010). Health system financing. Technical Brief series: Brief No 8. Available at: https://cdn.who.int/media/docs/default-source/health-financing/whr-2010-technical-brief-8.pdf?sfvrsn=1ef5aa64_3

[2] United Nations General Assembly. Resolution a/RES/74/2, political declaration of the high-level meeting on universal health coverage. New York, NY: United Nations General Assembly (2019).

[3] Cotlear D Nagpal S Smith O Tandon A Cortez R. Going universal: How 24 countries are implementing universal health coverage reforms from the bottom-up. Washington, DC: World Bank Group (2014).

[4] World Health Organization. Primary health care on the road to universal health coverage. 2019 Global monitoring report. Geneva, Switzerland: World Health Organization (2019).

[5] Bart C Maria PW. Declining subscriptions to the Maliando mutual health organisation in Guinea-Conakry (West Africa): what is going wrong? Soc Sci Med. (2003) 57:1205–19. doi: 10.1016/S0277-9536(02)00495-1, PMID: 12899905

[6] Shifa SH Shagufta HMA. The role of micro health insurance in providing financial risk protection in developing countries- a systematic review. BMC Public Health. (2016) 16:281. doi: 10.1186/s12889-016-2937-9, PMID: 27004824 PMC4802630

[7] Donfouet HPP Mahieu P-A. Community-based health insurance and social capital: a review. Heal Econ Rev. (2012) 2:5. doi: 10.1186/2191-1991-2-5, PMID: 22828204 PMC3402932

[8] WHO (2017). Community based health insurance: how can it contribute to progress towards UHC? Policy Brief. Available at: https://www.who.int/publications/i/item/WHO-HIS-HGF-PolicyBrief-17.3

[9] Wang H Pielemeier N. Community-based health insurance: an evolutionary approach to achieving universal coverage in low-income countries. J Life Sci. (2012) 6:320–9.

[10] Olaniran A Smith H Unkels R Bar-Zeev S van den Broek N. Who is a community health worker? – a systematic review of definitions. Glob Health Action. (2017) 10:1272223. doi: 10.1080/16549716.2017.1272223, PMID: 28222653 PMC5328349

[11] Moyehodie YA Fenta SM Mulugeta SS Agegn SB Yismaw E Biresaw HB. Factors associated with community based health insurance healthcare service utilization of households in South Gondar zone, Amhara, Ethiopia. A community-based cross-sectional study. Health Serv. Insights. (2022) 15:1–12. doi: 10.1177/11786329221096065, PMID: 35571582 PMC9092581

[12] Kusi A Enemark U Hansen KS Asante FA. Refusal to enrol in Ghana’s National Health Insurance Scheme: Is affordability the problem? Int J Equity Health. (2015) 14:2. doi: 10.1186/s12939-014-0130-2 25595036 PMC4300159

[13] Haile M Ololo S Megersa B. Willingness to join community-based health insurance among rural households of Debub Bench District, bench Maji zone, Southwest Ethiopia. BMC Public Health. (2014) 14:1–10. doi: 10.1186/1471-2458-14-591 24920538 PMC4074337

[14] Shigute Z Mebratie AD Sparrow R Alemu G. The effect of Ethiopia’s community-based health insurance scheme on revenues and quality of care. Int J Environ Res Public Health. 17:r8558. doi: 10.3390/ijerph17228558 PMC7698817 33218111

[15] HujSssien M Azage M. Barriers and facilitators of community-based health insurance policy renewal in low- and middle-income countries: a systematic review. Clinicoecon Outcomes Res. (2021) 13:359. doi: 10.2147/CEOR.S306855 34007193 PMC8123963

[16] Kellam Wollega Zonal Health Department. Annual report of 2021/2022 fiscal year. Dembi Dollo: Kellam Wollega Zonal Health Department.

[17] Eseta WA Lemma TD Geta ET. Magnitude and determinants of dropout from community-based health insurance among households in Manna District, Jimma zone, Southwest Ethiopia. Clin Outcomes Res. (2020) 12:747–60. doi: 10.2147/CEOR.S284702, PMID: 33364800 PMC7751608

[18] Gurung GB Panza A. Predictors of annual membership renewal to increase the sustainability of the Nepal National Health Insurance program: a cross-sectional survey. PLOS Glob Public Health. (2022) 2:e0000201. doi: 10.1371/journal.pgph.0000201, PMID: 36962197 PMC10021716

[19] Ashagrie B Biks GA Belew AK. Community-based health insurance membership dropout rate and associated factors in Dera District, Northwest Ethiopia. Risk Manag Healthc. (2020) 13:2835–44. doi: 10.2147/RMHP.S277804, PMID: 33304111 PMC7723227

[20] Dong H De Allegri M Gnawali D Souares A Sauerborn R. “Drop-out analysis of community-based health insurance membership at Nouna, Burkina Faso,” BurkinFaso. Health Policy Educ. (2009) 92:174–9. doi: 10.1016/j.healthpol.2009.03.013, PMID: 19394105

[21] Kaso AW Yohanis Y Debela BG Hareru HE. Community-based health insurance membership renewal rate and associated factors among households in Gedeo zone, southern Ethiopia. J Environ Public Health. (2022) 2022:1–11. doi: 10.1155/2022/8479834, PMID: 36225760 PMC9550414

[22] Atinga RA Abiiro GA Kuganab-Lem RB. Factors influencing the decision to drop out of health insurance enrolment among urban slum dwellers in Ghana. Tropical Med Int Health. (2015) 20:312–21. doi: 10.1111/tmi.12433, PMID: 25418283

[23] David M Shahed H Atanu M Koehlmoos TLP John D Panda PK. What factors affect voluntary uptake of community-based health insurance schemes in low- and middle-income countries? A systematic review and Meta-analysis. PLoS One. (2016) 11:1–31. doi: 10.1371/journal.pone.0160479 PMC5006971 27579731

[24] Tsega H Getu D Gashaw A. Determinants of community-based health insurance implementation in west Gojjam zone, Northwest Ethiopia: a community based cross sectional study design. BMC Health Serv Res. (2019) 19:544. doi: 10.1186/s12913-019-4363-z 31375108 PMC6679527

[25] Herberholz C. Determinants of voluntary national health insurance drop-out in eastern Sudan. Appl Health Econ Health Policy. (2016) 15:215–26. doi: 10.1007/s40258-016-0281-y, PMID: 27696328

[26] Iqbal M Chowdhury AH Mahmood SS Mia MN Hanifi SMA Bhuiya A. Socioeconomic and programmatic determinants of renewal of membership in a voluntary micro health insurance scheme: evidence from Chakaria, Bangladesh. Glob Health Action. (2017) 10:1287398. doi: 10.1080/16549716.2017.1287398, PMID: 28471332 PMC5496168

[27] Panda P Chakrabort A Raza W Bedi AS. Renewing membership in three community-based health insurance schemes in rural India. Health Policy Plan. (2016) 31:1433–44. doi: 10.1093/heapol/czw090, PMID: 27476500

[28] Zepre K Yassin F Tadesse B Tolossa O Hailemariam D Wondimu A. Factors influencing drop-out of households from community-based health insurance membership in rural districts of Gurage zone, southern Ethiopia: community based case-control study. Front Public Health. (2022) 10:925309. doi: 10.3389/fpubh.2022.925309, PMID: 36276388 PMC9581137

[29] Nshakira-Rukundo E Mussa EC Nshakira N Gerber N von Braun J. Determinants of enrolment and renewing of community-based health insurance in households with under-five children in rural South-Western Uganda. Int J Health Policy Manag. (2019) 8:593–606. doi: 10.15171/ijhpm.2019.49, PMID: 31657186 PMC6819630

[30] Mukangendo M Nzayirambaho M Hitimana R Yamuragiye A. Factors contributing to low adherence to community-based health insurance in rural Nyanza District, southern Rwanda. J Environ Public Health. (2018) 2018:1–9. doi: 10.1155/2018/2624591, PMID: 30662470 PMC6312613

[31] Khan JA Ahmed S. Impact of educational intervention on willingness-to pay for health insurance: a study of informal sector workers in urban Bangladesh. Health Econ Rev. (2013) 3:12. doi: 10.1186/2191-1991-3-12, PMID: 23628206 PMC3644264

[32] Ololo S Jirra C Hailemichael Y Girma B. Indigenous community insurance (IDDIRS) as an alternative health care financing in Jimma city Southwest Ethiopia. Ethiop J Health Sci. (2009) 19:53–60.

[33] Namomsa G. Assessing the practices and challenges of community-based health insurance in Ethiopia: the case of Oromia national regional state district of Gimbichu. Int J Ad Res. (2019) 7:734–54. doi: 10.21474/IJAR01/9095

[34] Asmamawu A. Community based health insurance in Ethiopia: enrollment, membership renewal, and effects on health service utilization. Seoul: Seoul National University (2018).

[35] Ozawa S Walker D. Trust in the context of community-based health insurance schemes in Cambodia: villagers’ trust in health insurer. Adv Health Econ Health Serv Res. (2009) 21:107–32. doi: 10.1108/S0731-2199(2009)0000021008, PMID: 19791701

[36] Awudu S. National health insurance scheme: predictors of card renewal among subscribers in the east Gonja district of Ghana. Ghana: University of Ghana (2016).

[37] Taddesse G Atnafu DD Ketemaw A Alemu Y. Determinants of enrollment decision in the community-based health insurance, north West Ethiopia: a case-control study. Glob Health. (2020) 16:4. doi: 10.1186/s12992-019-0535-1, PMID: 31906995 PMC6945744

